# Physicians’ Perceptions of Telemedicine Use During the COVID-19 Pandemic in Riyadh, Saudi Arabia: Cross-sectional Study

**DOI:** 10.2196/36029

**Published:** 2022-07-12

**Authors:** Sarah Idriss, Abdullah Aldhuhayyan, Ahmad Abdullah Alanazi, Walaa Alasaadi, Reem Alharbi, Ghadah Alshahwan, Mohammad Baitalmal, Wadi Alonazi

**Affiliations:** 1 Ministry of Health Riyadh Saudi Arabia; 2 Emergency Medical Services Saudi Red Crescent Authority Riyadh Saudi Arabia; 3 Respiratory Therapy Department King Abdulaziz Medical City Riyadh Saudi Arabia; 4 Health Administration Department King Saud University College of Business Riyadh Saudi Arabia; 5 Home Healthcare Department King Fahad Medical City Riyadh Saudi Arabia; 6 Quantitative Analysis Department King Saud University College of Business Administration Riyadh Saudi Arabia; 7 Health Administration Department King Saud University College of Business Administration Riyadh Saudi Arabia

**Keywords:** burnout, COVID-19, patient experience, perception, physicians, telemedicine, virtual

## Abstract

**Background:**

The term “telemedicine” refers to the use of communication technology to deliver health care remotely. The COVID-19 pandemic had substantial impacts on health care delivery from 2020 onward, and it was necessary to adapt high-quality care in a manner that limited the potential for viral exposure of both patients and health care workers. Physicians employed video, phone, and electronic written (e-consultation) visits, all of which provided quality of care comparable to that of face-to-face visits while reducing barriers of adopting telemedicine.

**Objective:**

This study sought to assess physicians’ perspectives and attitudes regarding the use of telemedicine in Riyadh hospitals during the COVID-19 pandemic. The main objects of assessment were as follows: (1) physicians’ experience using telemedicine, (2) physicians’ willingness to use telemedicine in the future, (3) physicians’ perceptions of patient experiences, and (4) the influence of telemedicine on burnout.

**Methods:**

This study employed SurveyMonkey to develop and distribute an anonymous 28-question cross-sectional survey among physicians across all specialty disciplines in Riyadh hospitals. A chi-square test was used to determine the level of association between variables, with significance set to *P*<.05.

**Results:**

The survey was distributed among 500 physicians who experienced telemedicine between October 2021 and December 2021. A total of 362 doctors were included, of whom 28.7% (n=104) were consultants, 30.4% (n=110) were specialists, and 40.9% (n=148) were residents. Male doctors formed the majority 56.1% (n=203), and female doctors accounted for 43.9% (n=159). Overall, 34% (n=228) agreed or somewhat agreed that the “quality of care during telemedicine is comparable with that of face-to-face visits.” Approximately 70% (n=254) believed that telemedicine consultation is cost-effective. Regarding burnout, 4.1% (n=15), 7.5% (n=27), and 27.3% (n=99) of the doctors reported feeling burnout every day, a few times a week, and a few times per month, respectively.

**Conclusions:**

The physicians had generally favorable attitudes toward telemedicine, believing that its quality of health care delivery is comparable to that of in-person care. However, further research is necessary to determine how physicians’ attitudes toward telemedicine have changed since the pandemic and how this virtual technology can be used to improve physicians’ professional and personal well-being.

## Introduction

### Background

“Telemedicine” refers to the use of IT in the provision of health care via electronic devices [1]. COVID-19, a highly infectious virus causing respiratory illness, has caused an ongoing global pandemic. Saudi Arabia experienced more than half a million infections and more than 7000 fatalities by June 2021 [1]. This outbreak has led to innovative practices such as Telemedicine among health providers. Thus, physicians have often opted to use telemedicine as an alternative to provide fast and safe care away from outpatient clinics, which require physical contact [2]. This research investigates physicians’ perceptions of telemedicine during the COVID-19 pandemic in Riyadh, Saudi Arabia.

Although various forms of telemedicine such as phone calls and electronic messaging have been used in Saudi Arabia for years, COVID-19 has led to the implementation and successful use of audiovisual technologies for patients across the country.

Previous limited evidence suggested that telemedicine could provide generally effective, comparable, and satisfactory quality of care as well as improvements in clinical outcomes [3]. However, there is a lack of larger studies of perception and attitude regarding patient-physician interactions, satisfaction with services and convenience of using telemedicine, preference for face-to-face communication, and support for technology infrastructure [4].

Furthermore, few studies have assessed whether the use of telemedicine affects physician well-being and burnout, as telemedicine theoretically provides more flexibility in terms of physician time and geographical location while performing virtual visits [5]. This paper is organized as follows: first, the research team examined the literature for studies related to physician perceptions of telemedicine; second, the method and the survey design were explained; third, the statistical analysis and results were presented; and finally, discussion, limitations, and conclusions and recommendations are presented.

### Physicians’ Experience Using Telemedicine

The United Kingdom’s National Health Service immediately implemented telemedicine as a substitute for face-to-face consultations during the COVID-19 pandemic [6]. Despite widespread unfamiliarity with telemedicine prior to the pandemic, there was a rapid rise in its usage among health care providers [6,7]. Moreover, web-based platforms became indispensable for boosting public health awareness and disseminating information about the pandemic [8].

The implementation of telemedicine, however, has encountered various barriers in Saudi Arabia. These issues include bureaucracy, lack of expertise, inadequate IT infrastructure, absence of guidelines, and insufficient institutional support [9]. Therefore, Saudi Arabia’s Ministry of Health must assess health care workers’ knowledge and perceptions of telemedicine to facilitate its future implementation while considering patient privacy and confidentiality concerns [10]. To the best of the authors’ knowledge, this is the first study to do so.

### Perceptions of Patient Experience

The success of any health care delivery system, including telemedicine, is heavily reliant on patient perceptions and satisfaction. Patients are the primary source of information that tells us whether health care is being delivered properly and whether the care they receive meets their expectations [11]. A study conducted by Power [12] on health care consumer satisfaction indicates that 66% of patients are either generally unaware that they can use telemedicine for consultations, or else it is not available to them. Among those who are aware, however, male patients, particularly those aged 18-59 years, are often satisfied with their telemedicine experiences [13]. Female patients, interestingly, are more likely to feel rather neutral toward telemedicine, that is, neither satisfied nor dissatisfied [13]. It is worth noting that almost half of the study’s respondents opined that the quality of telemedicine will never be on par with that of traditional in-person care [13]. Many telemedicine applications are available; however, due to a lack of knowledge about telemedicine technology, patients feel uncomfortable using and adapting to it. Therefore, user-friendly telemedicine apps should be developed to improve favorability among patients who feel that in-person care cannot be matched. Additionally, apps should be available in local languages to ensure that patients both learn about telemedicine and experience it in a positive and comfortable manner [14]. Moreover, policy makers around the world should work to boost patients’ awareness of telemedicine to ensure correct care during this pandemic and those to come [15].

In one study, most patients who had used telemedicine noted that the practice’s convenience and effectiveness helped them to seek treatment from remote areas and optimize their management of type 2 diabetes mellitus [15]. Another study found that the vast majority of patients believe that telemedicine had optimized their type 2 diabetes mellitus management; however, most of these patients noted that improvements could be made to aspects such as user-friendliness, interaction with medical team, and time required for recording or transferring data [16].

### Willingness to Use Telemedicine in the Future

Technological advancements have improved existing practices and paved the way for the expansion of telemedicine in the future. Such advancements in telemedicine have increased dependability, lowered costs, improved audiovisual quality, and emulated clinical settings more successfully (eg, by implementing virtual waiting rooms) [17]. There is significant potential in the scalability of telemedicine visits [18]. However, Florea et al [17] revealed that approximately 77% of professionals believe that continual training is essential for health care providers to stay up-to-date with advancements in telemedicine.

Of course, health care institutions are likely to face new ethical challenges arising from the use of telemedicine. For example, they must be capable of protecting patients’ private information against potential cyberattacks [17]. Telemedicine could also lead to a rise in malpractice claims stemming from a lack of appropriate guidelines and, in turn, problems with reimbursement [17]. Evidently, significant changes are necessary to fully incorporate telemedicine services into the health care landscape and to fully reap its benefits in advance of future pandemics [17].

Through technological advancements and appropriately oriented policy developments, telemedicine could become a sustainable mainstream solution for both public health emergencies and routine care [9]. Telemedicine may be a reasonable choice for physicians in the future if properly used by patients and if legal guidelines for telemedicine are implemented to address the aforementioned concerns [18].

### Effects of Telemedicine on Burnout

Burnout has been defined as a psychological syndrome involving emotional exhaustion, depersonalization, and a sense of reduced personal accomplishment [19]. A 2020 study conducted by Jha et al [19] assessed how COVID-19 has placed several physical and emotional stressors on physicians, which increased physician burnout. The demanding role of primary care physicians (PCPs) in pandemic mitigation measures has made them susceptible to psychological distress. Some PCPs fear being infected by COVID-19; this fear is exacerbated by a lack of personal protective equipment and extended shifts on the front line. All of these issues are in addition to PCPs’ existing anxiety stemming from a fast-paced, efficiency-oriented work environment. Many are concerned about errors of omissions and complaints of community residents. Additionally, the documentation process for reporting instances of COVID-19, which is perceived as time-consuming and not conducive to delivering high-quality care, is a source of frustration for most PCPs [20].

## Methods

### Ethics Approval

This study was approved by the institutional ethical committee of the King Fahad Medical City (IRB log:21-458).

### Study Design and Population

This paper presents a cross-sectional study conducted between October and November 2021 among physicians in Riyadh hospitals. A web link survey conducted through SurveyMonkey distributed an anonymous 28-question survey to 500 physicians. After obtaining institutional ethical committee approval, the questionnaire was sent through social media platforms such as WhatsApp, Twitter, and LinkedIn. A total of 362 participants returned the questionnaire, yielding a response rate of 60%. The questionnaire consisted of the following five sections: (1) demographic characteristics; (2) familiarity with telemedicine; (3) perceptions of patients’ experiences; (4) willingness to use telemedicine in the future; and (5) the effects of telemedicine on burnout. The responses were measured using a Likert scale. Each respondent’s current academic position (consultant, specialist, or intern), specialty, years of postresidency experience, age, sex, frequency of telemedicine use prior to the COVID-19 pandemic, and length of time using telemedicine were all variables of interest.

### Survey Design

The instrument tool used in this study was inspired by Malouff et al [21]. The tool was developed to evaluate the perception of physician perceptions and attitudes toward telemedicine. The anonymous survey consisted of 38 questions. The survey was developed in consultation with an expert panel with a consensus informed by elements from existing evidence and models, including the unified theory of acceptance and use of technology, Technology Acceptance Model 2, and diffusion of innovation frameworks [21].

### Statistical Analysis

The data were checked for completeness, and all errors were corrected. All of the variables were categorical; therefore, they are presented as frequencies and percentages. The responses of consultants, specialists, and residents—as well as those of men and women—were compared using chi-square tests. The analyses were performed at a 95% confidence interval using SPSS (v.23.0, IBM Corp). Physicians completed the survey including 28 items (sample item: “I find telemedicine has been easy to navigate and use”). The items were rated on a 5-point scale ranging from 1 (strongly agree) to 5 (strongly disagree). The Cronbach alpha for this study was .82, which is considered a good indication for internal consistency [22].

## Results

**Figure 1:**
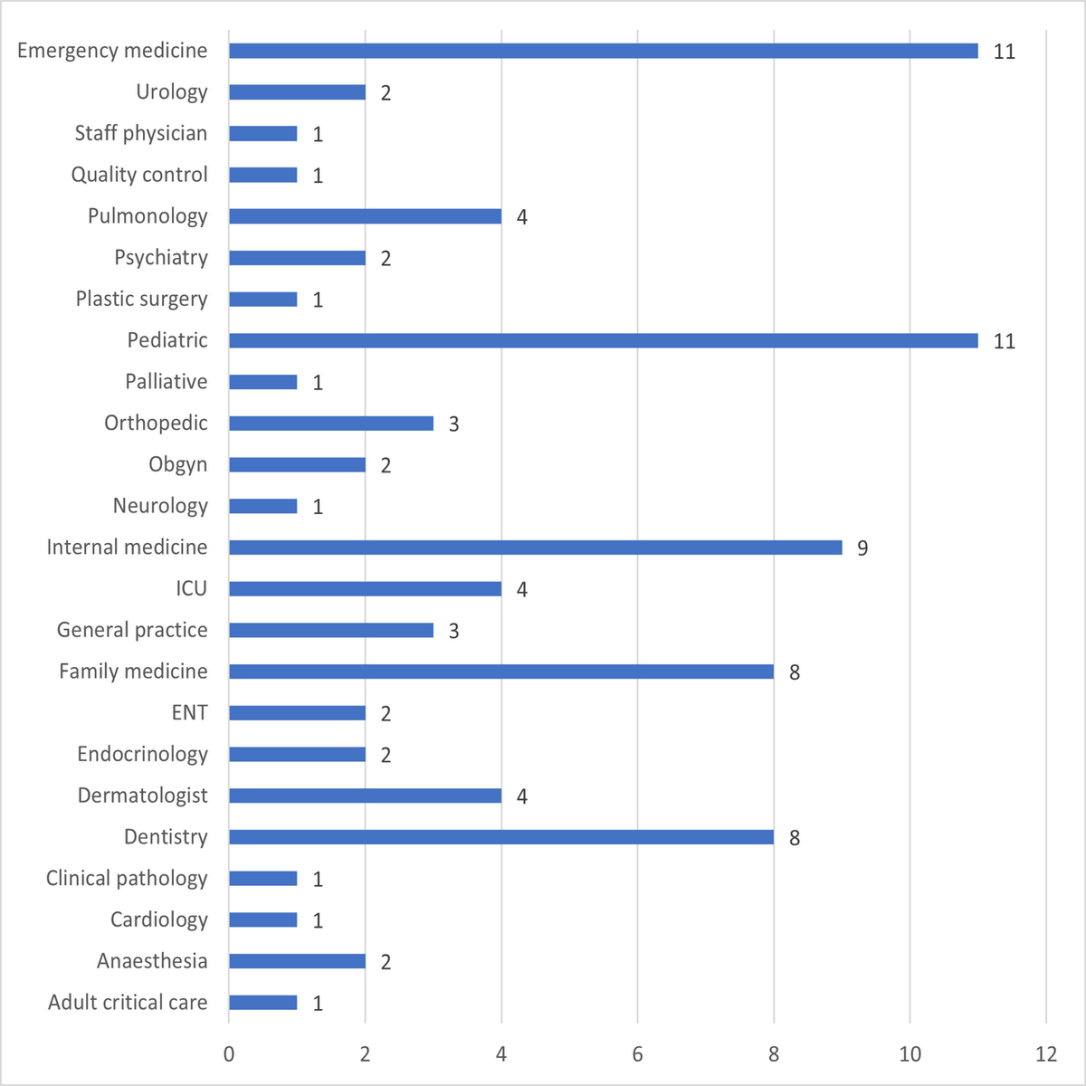
Specialty distribution. ENT: ear, nose, and throat; ICU: intensive care unit; Obgyn: obstetrics and gynecology.

The survey was sent to physicians who experienced telemedicine between October 2021 and December 2021. A total of 362 doctors were included, of whom 28.7% (n=104) were consultants, 30.4% (n=110) were specialists, and 40.9% (n=148) were residents. Male doctors formed the majority 56.1%, (n=203), and female doctors accounted for 43.9% (n=159). When asked about the frequency with which they use telemedicine during the pandemic, 41.4% (n=150) answered “frequently,” 26% (n=49) responded “occasionally,” and 32.6% (n=118) said “never” (Table 1). Only 25% (n=89) of doctors specified their specialty. Figure 1 shows the specialty distribution; although the physicians were from almost all specialties, they were most frequently from emergency medicine (n=10, 12%) and pediatrics (n=10, 12%).

Moreover, 34% (n=228) agreed or somewhat agreed that the “quality of care during telemedicine is comparable with that of face-to-face visits.” Approximately 70% (n=254) believed that telemedicine consultation is a cost-effective means of providing care relative to traditional face-to-face visits. Most of the doctors were skilled at delivering telemedicine 70% (n=163) and capable of independently solving technological issues during telemedicine visits 54% (n=195). Overall, the physicians felt that their patients view telemedicine positively; 68% (n=246) said that their patients felt comfortable using telemedicine, and 76% (n=273) stated that their patients would assert that telemedicine saves time. Regarding burnout, 4.1% (n=15), 7.5% (n=27), and 27.3% (n=99) of the doctors reported feeling burnout every day, a few times a week, and a few times per month, respectively.

**Figure 2:**
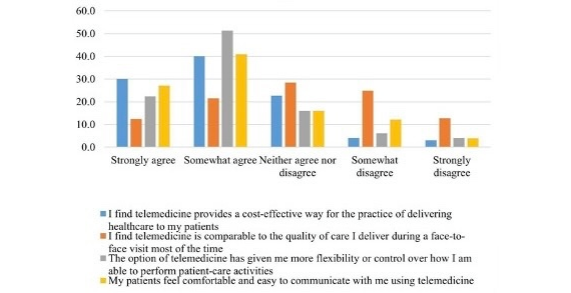
Physicians’ attitudes toward the quality and potential advantages of telemedicine.

**Figure 3:**
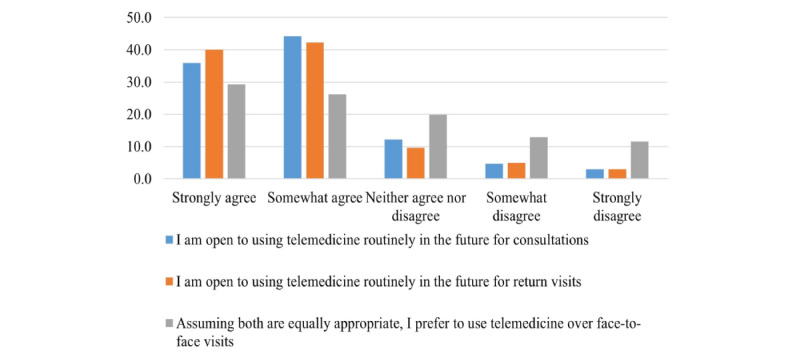
Physicians’ openness to using telemedicine after the COVID-19 pandemic.

The physicians’ responses to the Likert scale prompts are presented in Multimedia Appendix 1. In all, 31% (113/362) of the physicians agreed or somewhat agreed that “telemedicine’s quality of care is generally comparable to that which I deliver during face-to-face visits.” Approximately 55% (199/362) believed that telemedicine consultations are cost-effective relative to in-person visits. Moreover, 74% (268/362) believed that telemedicine gives them more flexibility or control over how they deliver patient care (Multimedia Appendix 1). Most of the physicians (254/362, 70%) asserted that they are skilled at telemedicine, and are capable of independently solving technological issues during telemedicine visits (195/362, 54%). Furthermore, most of the physicians felt that their patients view telemedicine in a positive manner; 68% (246/362) said that their patients felt comfortable using telemedicine, and 76% (275/362) said that their patients would assert that telemedicine saves time (Figures 2 and 3).

**Figure 4:**
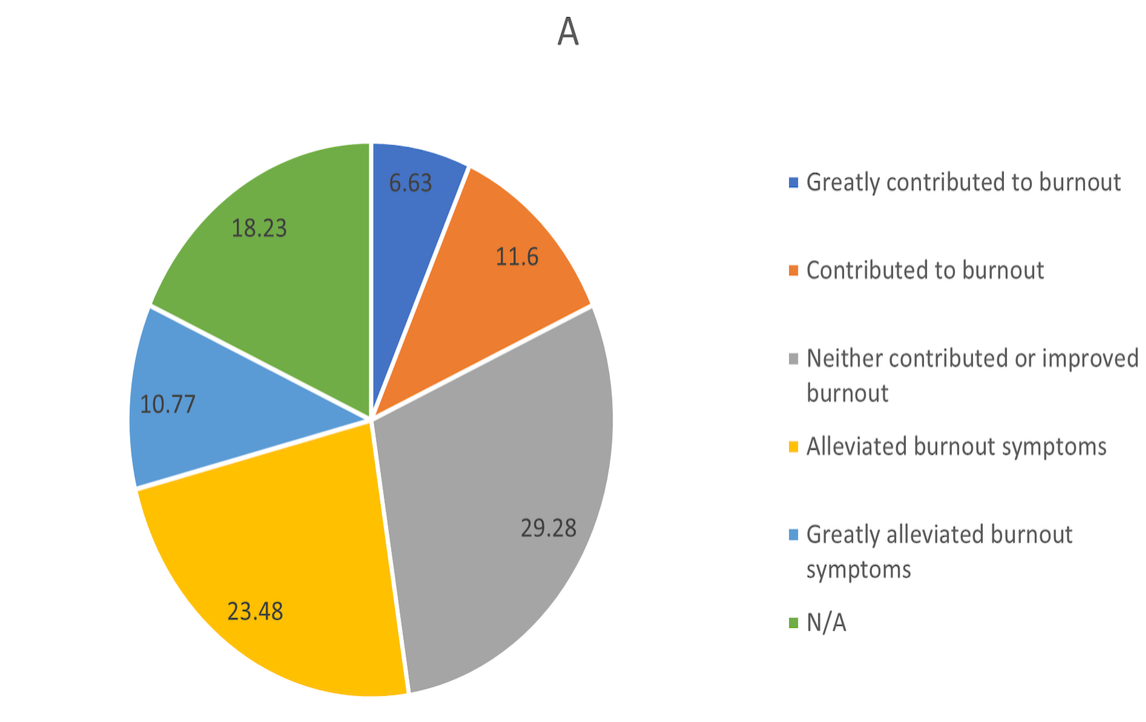
Responses regarding physician burnout and the influence of telemedicine: “What role has telemedicine played in your experience of burnout?” N/A: not applicable.

**Figure 5:**
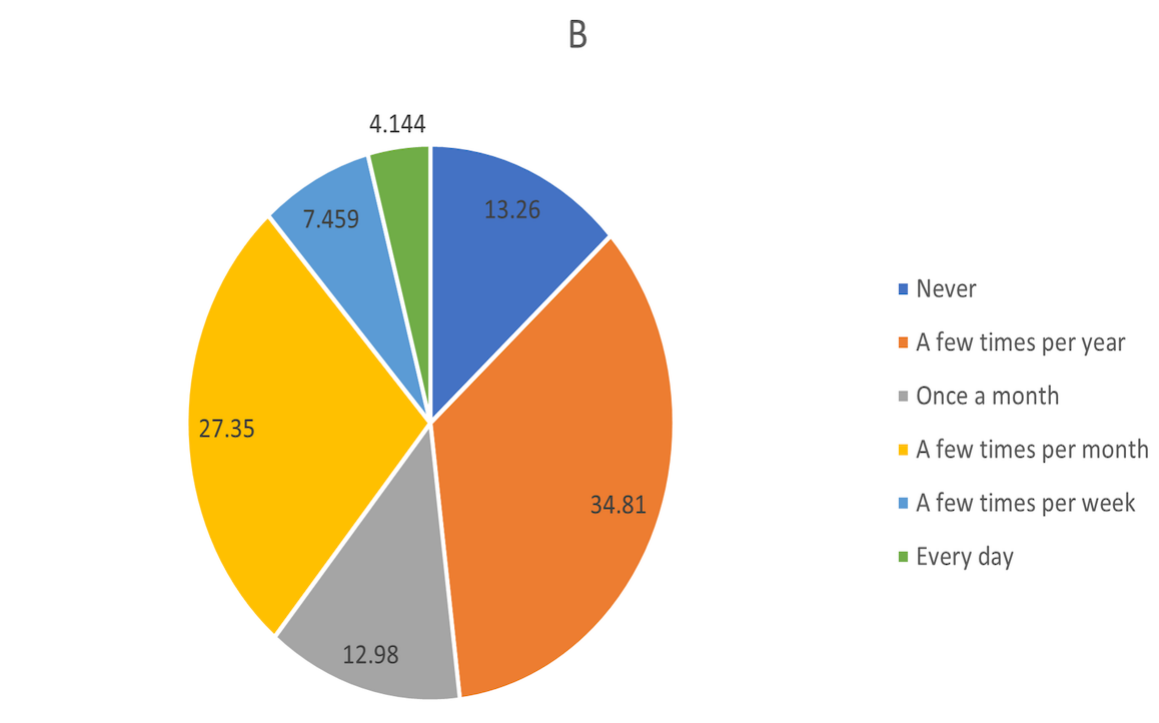
Responses regarding physician burnout and the influence of telemedicine: “I feel burnout from work”.

Regarding burnout, 4.1% (n=15), 7.5% (n=27), and 27.3% (n=99) of the physicians felt it every day, a few times per week, and a few times per month, respectively. When asked about the role of telemedicine in burnout, 23.5% (n=85) of physicians said that it alleviated their burnout symptoms. However, 11.6% (n=42) believed that telemedicine contributed to their burnout, and an additional 6.6% (n=24) thought that it substantially contributed to their burnout (Figures 4 and 5).

This study found no statistically significant difference between the burnout frequencies of men and women (*P*=.57). However, there was a statistically significant difference in burnout frequency between those in different academic positions (*P*=.002). Whereas 6.1% (9/148) of residents felt burnout every day, only 2.7% (3/110) of specialists and 2.9% (3/104) of consultants felt the same. Interestingly, 18.3% (19/104) of consultants asserted that telemedicine alleviated their burnout symptoms to a sizable degree, whereas only 7.3% (8/110) of specialists and 8.1% (12/148) of residents felt the same (*P*=.001).

**Table 1:** Distribution of all physicians by their characteristics and previous experiences with telemedicine.

Characteristics			Values, n (%)
**Current position**			
	Consultant	104 (28.7)	
	Specialist	110 (30.4)	
	Resident	148 (40.9)	
**Clinical experience following residency training (years)**			
	<5	142 (39.2)	
	5-10	114 (31.5)	
	11-20	77 (21.3)	
	21-30	25 (6.9)	
	>30	4 (1.1)	
**Current age (years)**			
	<30	114 (31.5)	
	31-40	140 (38.7)	
	41-50	85 (23.5)	
	51-60	23 (6.4)	
**Gender**			
	Male	203 (56.1)	
	Female	159 (43.9)	
**Frequency of telemedicine use before the COVID-19 pandemic**			
	Frequently (>1 time per month or >12 times per year)	150 (41.4)	
	Occasionally (1-12 times per year)	94 (26.0)	
	Never	118 (32.6)	
**Time spent using telemedicine in any medical capacity (years)**			
	<1	154 (42.5)	
	2-3	106 (29.3)	
	>3	102 (28.2)	

## Discussion

### Principal Findings

Our data suggest that physicians in Riyadh have adapted to the implementation of telemedicine. Most of the physicians assessed in this study, regardless of their specialty, considered telemedicine to be easy to navigate and use. Furthermore, almost all respondents were open to using telemedicine for routine consultations and follow-ups even after the pandemic ends; in fact, more than half of them actively preferred telemedicine to in-person visits. The findings of this work align with those of previous studies. For example, Gillman-Wells et al [6] found that 70% of plastic surgeons surveyed in the United Kingdom have embraced the use of telemedicine. A survey by Srinivasan et al [7] found that personnel at Stanford University’s primary care clinics strongly believe that telemedicine visits should be an ongoing element of health care delivery after the pandemic.

There are several potential reasons for why physicians are willing to adapt to this new technology, including cost-effectiveness, time efficiency for physicians and patients alike, as well as flexibility in scheduling telemedicine visits, all of which may improve physicians’ quality of life [21]. These advantages balance out concerns over lower quality of care. According to a study conducted during the first year of the pandemic, one major shortfall of telemedicine that physicians should be aware of is the absence of physical examination [23].

As mentioned in the literature review, the cost-effectiveness of telemedicine relative to traditional in-person care is a significant driver of positive attitudes toward telemedicine; in this study, 70% (254/362) of the physicians agreed that it is a cost-effective way to deliver health care (in a study by the Mayo Clinic, 80% of the respondents agreed with this sentiment) [21]. To assess the verity of this perception, a study evaluated postoperative visit costs and found that patients who used telemedicine services saved an average of US $888 per return visit (increasing to US $1501 when accounting for travel and accommodation costs). The authors reported savings of US $256 per visit, even for patients who did not require accommodation [24]. Furthermore, the Pediatric Cardiology Service at the Coimbra University Hospital Center analyzed telemedicine use in Portugal since 1998 and found that it had saved the country’s health care system about EUR €1.1 million (US $1.3 million) overall, equating to savings of approximately EUR €419 (US $500) per patient [25]

Telemedicine also offers considerable efficiency for both physicians and patients. In this study, nearly three-quarters of the respondents agreed that telemedicine enhanced flexibility. Telemedicine visits can be conducted anywhere (including from home), and this ability helps physicians to balance their professional and personal needs, particularly during the COVID-19 pandemic. According to a study conducted by Chaudhry et al [26], telemedicine visits may also reduce some time-consuming activities that are common at clinics, such as waiting for rooms to become available, checking patients in, and moving patients from one room to another. Orthopedic patients reported that telemedicine saved them time, both when including (180 minutes) and excluding (17 minutes) travel time.

Regardless of the benefits that many physicians derive from telemedicine, more than one-third of this survey’s respondents did not agree that telemedicine is equivalent to in-person visits in terms of quality of care. This finding aligns with those of previous studies. Although telemedicine can be efficient and cost-effective, physicians lose the ability to conduct physical examinations, which are often crucial in order to meet patients’ needs and deliver appropriate preventative care. Furthermore, the patient-physician relationship largely depends on face-to-face visits. However, because telemedicine is even more common at the time of writing this paper compared to when this study was conducted, further surveys are necessary to understand physicians’ concerns and their relative importance.

Indeed, the most common concerns over telemedicine pertained to an inability to provide care on the same level as traditional in-person care. Zhang et al [5] considered this concern when examining the Memorial Sloan Kettering Cancer Center during the COVID-19 pandemic. They found that 92% of the center’s radiation oncology visits were conducted via telemedicine at the peak of the pandemic [5]. Overall, 71% of the providers reported no difference in their ability to treat cancer appropriately, and 55% of the patients reported no difference in their overall visit quality [5].

Although our study did not directly examine patients’ experiences of differences between virtual and in-person consultations regarding lab tests, imaging exams, and prescribed medications, a study on Stanford’s ClickWell Care clinic evaluated practice patterns for both telemedicine and in-person visits. It found no difference in laboratory tests, imaging tests, or prescriptions ordered between virtual and in-person visits for 17 of the most common diagnoses. However, overall, there were more laboratory and imaging tests ordered following in-person visits for all diagnoses; this increase may have affected general medical examinations [27].

Another widely reported concern when using telemedicine was the absence of a physical evaluation, which remains an essential element of follow-up care, mainly when assessing adverse posttherapy events for patients with physical disabilities [28]. To counter this limitation, some physicians are working to develop evaluations that can function via telemedicine, such as a neurosurgical spine examination that can be conducted remotely [29]. Early evidence on the feasibility and comparability of such examinations is promising. In addition, Laskowski et al [28] developed a specific set of guidelines to enhance evaluations of the musculoskeletal system when performing virtual examination.

Physical examinations are also correlated with patient satisfaction, which is critical in telemedicine because cost and time savings are meaningless if patients do not believe that they are receiving high-quality care [21]. Although this study did not directly survey patients, approximately 75% of the physicians felt that their patients were at ease communicating with them via telemedicine, with half agreeing that their patients found the technology easy to use and comparable in quality of care with face-to-face visits. These findings align with those of previous studies. For example, a study by Elawady et al [30] found that 73% of the physician respondents felt that their patients understood their medical conditions and the corresponding recommendations given to them over the phone. In addition, physicians were asked whether videoconference consultation would improve patient care over telephone consultation alone, and 70% of the respondents agreed that it would [30]. Moreover, according to a meta-analysis conducted by Chaudhry et al [26], there were no differences in surgeon satisfaction or patient-reported outcome measurements when comparing telemedicine visits with in-person visits [26].

Although patient satisfaction with telemedicine is critical, reduction of burnout among physicians by boosting flexibility in care delivery is one potential benefit of telemedicine. Telemedicine may reduce transportation time, granting physicians more time for sleep, family life, and social activities, all of which are key factors in avoiding burnout [21]. This study found that more than one-third of the surveyed physicians’ burnout symptoms were alleviated or substantially alleviated due to telemedicine. However, it is important to note that these figures may have been influenced by other stress factors related to the COVID-19 pandemic [21]. Further studies may be needed to evaluate whether—and to what degree—burnout can be mitigated through the postpandemic use of telemedicine.

According to Malouff et al [21], looking ahead, telemedicine will be an essential element of post–COVID-19 crises. Virtual reality has been proposed as a means of improving feelings of physical presence during examinations to compensate for the lack of physical presence during virtual visits [21]. In addition, telemedicine may be beneficial for people who are fearful of visiting clinics or hospitals; patients with anxiety or depression may prefer telemedicine to in-person visits. Finally, telemedicine may offer an opportunity for underrepresented populations to participate in clinical trials because follow-ups and toxicity supervision can be conducted virtually [27].

### Limitations

Despite this study’s finding that physicians’ perceptions of telemedicine are generally favorable, it comes with certain limitations. For instance, this was a survey-based study, meaning that it is subject to the typical limitations of survey-based evaluations, including incomplete responses and a low response rate. It was unable to precisely determine response rates because the survey link was partially distributed through social media platforms. Another major limitation of this study is that it does not directly survey patients. Furthermore, the results of this study are not generalizable to a wider health care population, given its small sample size and the sample size of the subgroups that were examined when comparing survey responses.

### Conclusions and Recommendations

This study shows that physicians in Riyadh, Saudi Arabia, have generally favorable attitudes toward the adoption of telemedicine because they believe that the quality of care delivered using telemedicine is comparable to that delivered using traditional methods. However, further research is necessary to correctly assess how the COVID-19 pandemic influenced physicians’ attitudes toward telemedicine and how telemedicine can be used to advance care delivery and improve patient outcomes in the future.

## Appendix

Multimedia Appendix 1 Physicians’ thoughts, attitudes, and capacities pertaining to telemedicine.

## Abbreviations

PCP: primary care physicians
