# Supplementary material for: Physicians’ Perceptions of Telemedicine Use During the COVID-19 Pandemic in Riyadh, Saudi Arabia: Cross-sectional Study
Source: JMIR Form Res. 2022 Jul 12;6(7):e36029. doi: 10.2196/36029 (PMC9278404; doi:10.2196/36029)
Supplement: Multimedia Appendix 1 [file formative_v6i7e36029_app1.docx]

**Multimedia Appendix 1.** Physicians’ thoughts, attitudes, and capacities pertaining to telemedicine.

| Questions and responses | | *n* | % |
| --- | --- | --- | --- |
| Physicians’ Experience Using Telemedicine | | | |
| I find that telemedicine is easy to navigate and use. | | | |
|  | Strongly agree | 110 | 30.4 |
|  | Somewhat agree | 157 | 43.4 |
|  | Neither agree nor disagree | 49 | 13.5 |
|  | Somewhat disagree | 26 | 7.2 |
|  | Strongly disagree | 20 | 5.5 |
| I feel skilled at using telemedicine. | | | |
|  | Strongly agree | 98 | 27.1 |
|  | Somewhat agree | 154 | 42.5 |
|  | Neither agree nor disagree | 69 | 19.1 |
|  | Somewhat disagree | 24 | 6.6 |
|  | Strongly disagree | 17 | 4.7 |
| I find it easy to solve technological issues during telemedicine consultations. | | | |
|  | Strongly agree | 49 | 13.5 |
|  | Somewhat agree | 146 | 40.3 |
|  | Neither agree nor disagree | 95 | 26.2 |
|  | Somewhat disagree | 54 | 14.9 |
|  | Strongly disagree | 18 | 5.0 |
| I find that telemedicine constitutes a cost-effective means of delivering health care to my patients. | | | |
|  | Strongly agree | 109 | 30.1 |
|  | Somewhat agree | 145 | 40.1 |
|  | Neither agree nor disagree | 82 | 22.7 |
|  | Somewhat disagree | 15 | 4.1 |
|  | Strongly disagree | 11 | 3.0 |
| I find that telemedicine’s quality of care is generally comparable to that which I deliver during face-to-face visits. | | | |
|  | Strongly agree | 45 | 12.4 |
|  | Somewhat agree | 78 | 21.5 |
|  | Neither agree nor disagree | 103 | 28.5 |
|  | Somewhat disagree | 90 | 24.9 |
|  | Strongly disagree | 46 | 12.7 |
| I feel that I have sufficient IT support for telemedicine visits in my practice. | | | |
|  | Strongly agree | 79 | 21.8 |
|  | Somewhat agree | 149 | 41.2 |
|  | Neither agree nor disagree | 72 | 19.9 |
|  | Somewhat disagree | 37 | 10.2 |
|  | Strongly disagree | 25 | 6.9 |
| Telemedicine has given me more flexibility/control over how I perform patient-care activities. | | | |
|  | Strongly agree | 81 | 22.4 |
|  | Somewhat agree | 186 | 51.4 |
|  | Neither agree nor disagree | 58 | 16.0 |
|  | Somewhat disagree | 22 | 6.1 |
|  | Strongly disagree | 15 | 4.1 |
| The telemedicine system in my practice needs technological improvements. | | | |
|  | Strongly agree | 102 | 28.2 |
|  | Somewhat agree | 96 | 26.5 |
|  | Neither agree nor disagree | 133 | 36.7 |
|  | Somewhat disagree | 26 | 7.2 |
|  | Strongly disagree | 5 | 1.4 |
| Future adaptability to using telemedicine | | | |
| I am open to using telemedicine routinely for consultations in the future. | | | |
|  | Strongly agree | 130 | 35.9 |
|  | Somewhat agree | 160 | 44.2 |
|  | Neither agree nor disagree | 44 | 12.2 |
|  | Somewhat disagree | 17 | 4.7 |
|  | Strongly disagree | 11 | 3.0 |
| I am open to using telemedicine routinely for return visits in the future. | | | |
|  | Strongly agree | 145 | 40.1 |
|  | Somewhat agree | 153 | 42.3 |
|  | Neither agree nor disagree | 35 | 9.7 |
|  | Somewhat disagree | 18 | 5.0 |
|  | Strongly disagree | 11 | 3.0 |
| Learning to use telemedicine has positively influenced my professional growth. | | | |
|  | Strongly agree | 90 | 24.9 |
|  | Somewhat agree | 167 | 46.1 |
|  | Neither agree nor disagree | 73 | 20.2 |
|  | Somewhat disagree | 26 | 7.2 |
|  | Strongly disagree | 6 | 1.7 |
| Assuming that both are equally appropriate, I prefer telemedicine to face-to-face visits. | | | |
|  | Strongly agree | 106 | 29.3 |
|  | Somewhat agree | 95 | 26.2 |
|  | Neither agree nor disagree | 72 | 19.9 |
|  | Somewhat disagree | 47 | 13.0 |
|  | Strongly disagree | 42 | 11.6 |
| I plan to earn further medical education credits to enhance my knowledge of telemedicine. | | | |
|  | Strongly agree | 83 | 22.9 |
|  | Somewhat agree | 173 | 47.8 |
|  | Neither agree nor disagree | 71 | 19.6 |
|  | Somewhat disagree | 28 | 7.7 |
|  | Strongly disagree | 7 | 1.9 |
| Perception about patients’ experience | | | |
| My patients feel that telemedicine is easy to navigate and use. | | | |
|  | Strongly agree | 46 | 12.7 |
|  | Somewhat agree | 155 | 42.8 |
|  | Neither agree nor disagree | 96 | 26.5 |
|  | Somewhat disagree | 48 | 13.3 |
|  | Strongly disagree | 17 | 4.7 |
| My patients find it easy to solve technological issues during telemedicine visits. | | | |
|  | Strongly agree | 41 | 11.3 |
|  | Somewhat agree | 48 | 13.3 |
|  | Neither agree nor disagree | 158 | 43.6 |
|  | Somewhat disagree | 92 | 25.4 |
|  | Strongly disagree | 23 | 6.4 |
| My patients find using telemedicine for consultations to be more cost-effective than face-to-face visits. | | | |
|  | Strongly agree | 54 | 14.9 |
|  | Somewhat agree | 146 | 40.3 |
|  | Neither agree nor disagree | 110 | 30.4 |
|  | Somewhat disagree | 27 | 7.5 |
|  | Strongly disagree | 25 | 6.9 |
| My patients find telemedicine’s quality of care to be comparable to that which they receive during face-to-face visits. | | | |
|  | Strongly agree | 40 | 11.0 |
|  | Somewhat agree | 73 | 20.2 |
|  | Neither agree nor disagree | 89 | 24.6 |
|  | Somewhat disagree | 115 | 31.8 |
|  | Strongly disagree | 45 | 12.4 |
| My patients feel comfortable and easily communicate with me when using telemedicine. | | | |
|  | Strongly agree | 98 | 27.1 |
|  | Somewhat agree | 148 | 40.9 |
|  | Neither agree nor disagree | 58 | 16.0 |
|  | Somewhat disagree | 44 | 12.2 |
|  | Strongly disagree | 14 | 3.9 |
| My patients feel that using telemedicine saves time. | | | |
|  | Strongly agree | 157 | 43.4 |
|  | Somewhat agree | 119 | 32.9 |
|  | Neither agree nor disagree | 60 | 16.6 |
|  | Somewhat disagree | 20 | 5.5 |
|  | Strongly disagree | 6 | 1.7 |
| The effect of telemedicine on burnout | | | |
| I feel burned out from work. | | | |
|  | Never | 48 | 13.3 |
|  | A few times per year | 126 | 34.8 |
|  | Once a month | 47 | 13.0 |
|  | A few times per month | 99 | 27.3 |
|  | A few times per week | 27 | 7.5 |
|  | Every day | 15 | 4.1 |
| What role has telemedicine played in your experience of burnout? | | | |
|  | Greatly contributed to burnout | 24 | 6.6 |
|  | Contributed to burnout | 42 | 11.6 |
|  | Neither exacerbated nor improved burnout | 106 | 29.3 |
|  | Alleviated burnout symptoms | 85 | 23.5 |
|  | Greatly alleviated burnout symptoms | 39 | 10.8 |
|  | N/A | 66 | 18.2 |
